# Gaps and opportunities in mental health support for young people: a process evaluation of a multi component intervention

**DOI:** 10.3389/fpubh.2025.1684562

**Published:** 2025-11-05

**Authors:** Jessica T. Oha, Gemma Bridge, Chris Flood, Catherine L. Jenkins, Charlotte Taylor-Page, Susie Sykes, Jane Wills, Wendy York, Patrick Callaghan, Paula Reavey

**Affiliations:** ^1^College of Health and Life Sciences School of Applied and Health Sciences, London South Bank University, London, United Kingdom; ^2^College of Health and Life Sciences School of Nursing and Midwifery, London South Bank University, London, United Kingdom; ^3^Central London Community Healthcare NHS Trust, London, United Kingdom; ^4^Mental Health Nursing Department, School of Health and Medical Sciences, London, United Kingdom

**Keywords:** youth mental health, life transitions, community-based intervention, cost-effectiveness, wellbeing outcomes, mental health services

## Abstract

**Background:**

Young people aged 16–25 are reporting rising rates of poor mental health, exacerbated by service gaps. Key life transitions such as moving from school to college, or into the workforce can increase vulnerability.

**Method:**

A mixed-methods evaluation was conducted of a multi-component, mental health intervention in the East of England. The evaluation aimed to: (1) Assess the fidelity, dose, and reach of the intervention; (2) Understand the mechanisms of impact and how change was generated; (3) Explore the influence of context, including dynamic relationships between those involved in delivering and accepting support, settings, and service delivery models; and (4) Assess if the intervention offered good value for money. Semi-structured interviews were held with local public health staff (*n* = 3), and an intervention lead. A focus group was conducted with intervention leads (*n* = 3). Photo production interviews were held with young people (*n* = 10). Quantitative outcomes were explored through pre- and post- questionnaires (*n* = 34), and pre-post intervention assessment of young people’s wellbeing, and satisfaction with the intervention, using the DIALOG (*n* = 34) and Wellbeing star measures (*n* = 37). Value for money was assessed using commissioned, in-house cost data and qualitative insights.

**Results:**

The intervention demonstrated positive outcomes in life satisfaction and functional wellbeing for young people, with young people engaging across the intervention components (*n* = 82, CC Hubs, *n* = 74, WN, *n* = 53, BR). The most cost-effective components were Upskilling the Workforce and Wellbeing Navigator support. Third spaces, and creative methods, fostered engagement and acceptability among young people.

**Conclusion:**

Integrated, co-produced, and place-based approaches can support young people’s mental health needs during life transitions. Investment in local partnerships and youth-centred design is important.

## Highlights

- What is known? Young people aged 16–25 face significant mental health challenges during life transitions, yet current services are often inaccessible, fragmented, and limited by rigid thresholds.
- What is new? This evaluation offers early insights into the implementation and cost-effectiveness of a co-produced, multi-component intervention spanning education, community, and workforce settings.
- What is significant for clinical practice? Flexible, relationship-based mental health support in community settings may enhance engagement among underserved young people, though long-term impact requires further study.

## 1 Introduction

Globally, mental health conditions are the leading cause of disability in young people, with suicide the second leading cause of death among 15–29-year-olds (1). In 2023, 23.3% of UK 17-19-year-olds and 21.7% of 20-25-year-olds had a diagnosed mental health condition (2). Meanwhile, mental health referrals are increasing, with a 50% increase from 821,734 in 2021/22 to 1,288,653 in 2022/23 (3).

The transition from childhood to adulthood marks the peak period onset of mental health conditions (4) exacerbated by identity formation, growing autonomy, and responsibilities, combined with leaving education and entering employment (1). Young people can also face challenges during this time with social network disruption (5).

Mental health challenges are not felt equally across young people. There are disparities in mental health experience across socioeconomic status, geography, and dis(ability) (6), ethnicity (7, 8), gender and sexuality (2, 9).

Current mental health service provision in the UK is fragmented, with rigid age cut-offs, poor coordination across services, and complex referral pathways (10, 11). In 2023/24, young people faced an average waiting time for treatment of 389 days (12). Compounding this, are young people’s concerns relating to stigma, cultural beliefs about mental health and support seeking, and the assumption that they should be able to manage on their own (13–15).

Interest in place-based approaches that address the complex, interrelated causes of ill health, and promote wellbeing across physical, psychological, and social dimensions is growing (16). This includes the role of non-clinical settings—such as communities, educational institutions, and workplaces—in supporting young people’s mental health (17–19).

### 1.1 The intervention and evaluation

In this paper we share the findings of a mixed methods evaluation of a placed based intervention. The intervention was co-designed with young people recruited from a local Health Watch and from the local child and young person mental health service through a series of focus groups. The intervention was delivered by local organisations (*n* = 3) and educational settings (*n* = 7) in the East of England. The intervention included four components which were designed to run simultaneously, supporting young people both within education settings, and in the community, and the public health and education workforce: (1) ‘Building Resilience’ (BR)—a whole-school/college approach using co-developed mental health action plans, and communities of practice (CoPs) developed for peer learning and collaboration among school staff; (2) ‘Wellbeing Navigators’ (WN)—8 weeks of one-to-one mental health support offered to 16–25-year-olds, delivered by MIND in community settings; (3) ‘Community Collaboration Hubs’ (CC hubs)—inclusive, cross-sector creative spaces for mental health support, including a youth theatre, a therapeutic writing programme and a rural community’s charity offering a youth to adult transition programme (one Wellbeing Navigator was based at each CC hub); and (4) ‘Upskilling the Workforce’ (UW)—training for education and Voluntary, Community, Faith, and Social Enterprise sector (VCFSE) professionals to better support the mental health of young people most in need. The intervention targeted vulnerable 16–25-year-olds, including neurodiverse, LGBTQ+, and ethnic minority young people, youth carers, and care leavers. Target groups were identified using GP records, local government data, Schools and Students Health Education Unit (SHEU) surveys, NHS referrals, and third-sector insights (see (20)).

### 1.2 Aims of the evaluation

Considering the UK Medical Research Council’s guidance on evaluating complex interventions (21), the evaluation aimed to: (1) Assess the fidelity (how closely the intervention was delivered as intended), dose (the extent to which the intervention was fully implemented), and reach (the number and spread of young people that engaged) with the intervention; (2) Understand the mechanisms of impact and how change was generated; (3) Explore the influence of context, including dynamic relationships between those involved in delivering and accepting intervention support, the settings, and service delivery models; and to (4) Explore cost effectiveness by conducting a cost analysis to assess if the intervention offered good value for money.

## 2 Methods

### 2.1 Evaluation design

**Figure 1:**
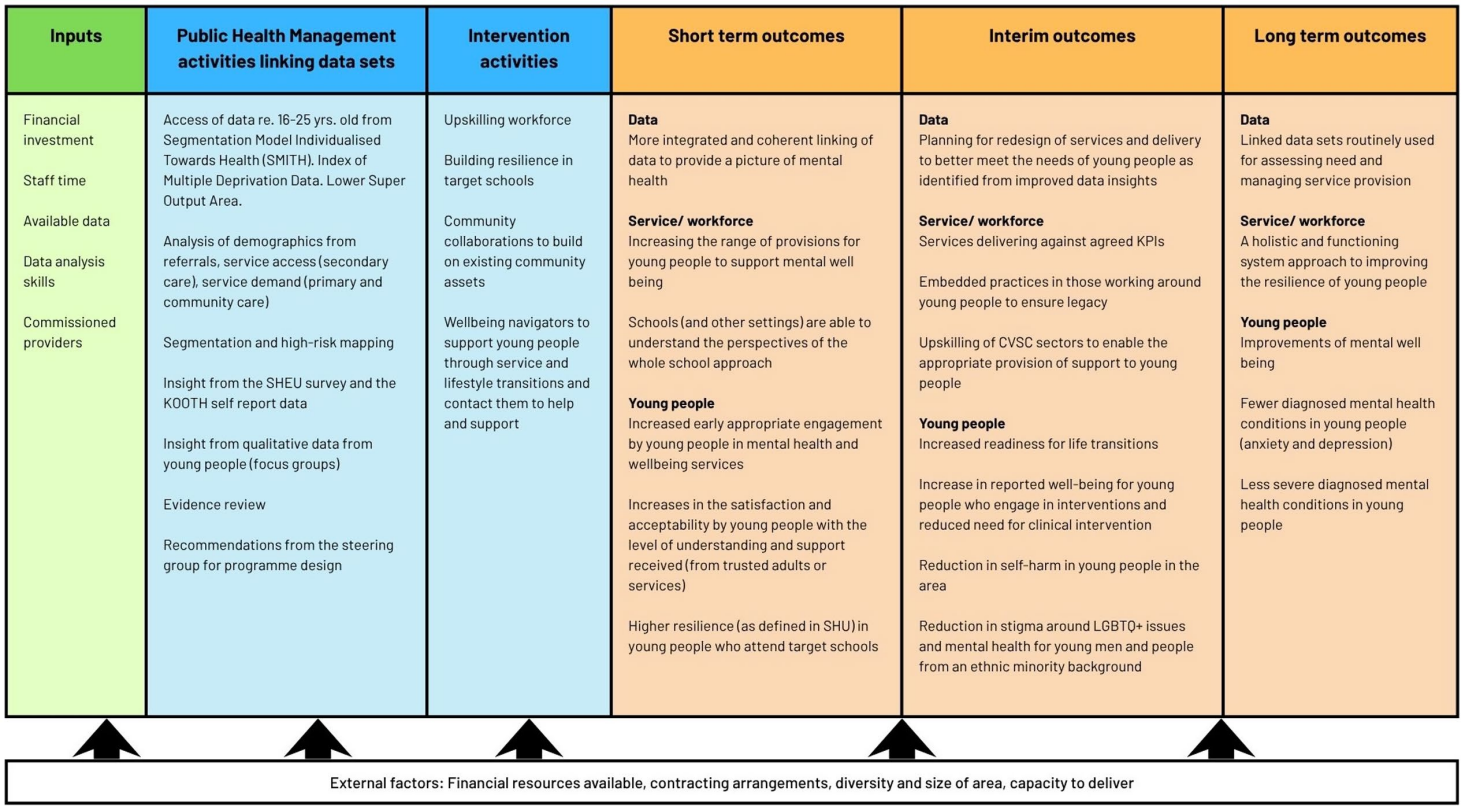
Logic model for the evaluation.

The evaluation employed a parallel mixed-methods design informed by a co-produced logic model (see Figure 1), built using Context, Mechanism, and Outcome (CMO) configurations to explore how intervention components generate short, medium and long-term change (44). Researchers, VCFSE representatives, public health specialists, and an adult public contributor co-developed the model through three online workshops. A PPI group (including 6 young people from the intervention area) was held between workshop 2 and 3, giving young people the opportunity to feed into the logic model development and development of the wider evaluation plan. The young people highlighted the importance of a young person centred approach to the evaluation, challenges with building trust between young people and adults (including teachers and parents), and the diversity among young people—which the young people said needed to be considered in the evaluation design and any outputs.

### 2.2 Data collection

The evaluation was supported by a panel of Public Contributors comprising local young people (*n* = 6), aged 16–25, who co-designed research and recruitment tools, reviewed participant materials, and informed the development and dissemination processes. All data were collected and analysed between July 2023 and March 2024 (see Table 1).

**Table 1:** Data collected to explore each research aim.

Evaluation phase	Aim(s)	Data source(s) and participant numbers (n)
Intervention delivery	To understand implementation of the intervention (fidelity, dose, reach) Exploring Mechanisms of Impact Assess Cost-Effectiveness	Qualitative interviews with local government public health staff (*n* = 2) Quantitative service level KPI data (e.g., number of young people referred, outcome of referrals, demographics of young people, duration of engagement) Qualitative service level KPI data Quantitative commissioned unit cost data and any available in-house costs One focus group discussion with intervention workstream leads (*n* = 3) and a member of local government public health staff (*n* = 1) One interview with an intervention workstream lead (*n* = 1)
Lived experience of intervention	Exploring Mechanisms of Impact	Qualitative interviews, using photo production, with young people (*n* = 10) Qualitative interviews with service delivery staff (*n* = 3).

#### 2.2.1 Intervention outcomes (KPI data) and cost effectiveness

A secondary analysis of quantitative Key Performance Indicator (KPI) data was conducted using data provided by local government and commissioned services (see Appendix A). This included pre- and post-intervention data, total budgets, component-level costs, and staffing. Data were securely shared and analysed in Excel under a data-sharing agreement.

Qualitative KPI and cost effectiveness data including impact proformas, a focus group with intervention leads and a public health practitioner (*n* = 4), and an interview with an additional intervention lead (*n* = 1) were collected to contextualise findings. Focus groups and interviews were conducted online using MS Teams using structured guides covering delivery, perceived outcomes, and sustainability (see Table 2).

**Table 2:** Interview and focus group topics with component leads, upskilling the workforce attendees and young people.

Participant group	Topic	Example questions
Intervention component leads	Intervention design	Have you had any experience in using a ‘community collaborations approach’?
	Intervention implementation	What have been the main milestones in implementing your intervention stream? What changes, if any, did you make to your intervention streams once they started?
	Seeing the big picture	How do you see your respective intervention streams contributing overall to YP’s mental health? What data would need to be measured / joined up to better support assessing need / managing service provision / re-design of services?
Upskilling the workforce attendees	Application of the intervention	Please describe an example of how you have seen the intervention applied.
	Evaluation of the intervention	How would you characterise the intervention—any key ingredients you would point to? Are there any difficulties you have encountered with this approach? In practice, do you think the intervention has/has not worked? How has it been for you working with this group of young people who are facing quite significant challenges with their mental health?
Young people	Photo discussion	Please can you describe each of the photos that you have brought with you today? Has anything changed about this space/person/situation since you became aware of your mental health challenges?
	Reason for attending the service	Can you start by describing what brought you to the intervention in the first place? What happened in the lead up to that?
	Positives and negatives about the service	Did you come across any difficulties with it? How did you get on with the person you saw? Did you feel they understood what you were going through?
	Changes because of the service	During the intervention, did you notice any changes to how you were engaging with school/college? Did you notice any other changes in your social life? Do you think the intervention has helped you manage things differently?

#### 2.2.2 Lived experiences of the intervention: visual-interviews with young people

All young people involved in the intervention were eligible for interviews (*n* = 82 from CC Hubs, *n* = 74 from WN, *n* = 53 from BR). Recruitment was via a co-designed poster, developed with the public and patient involvement (PPI) group and shared through service leads. Due to recruitment challenges, only young people from the CC Hubs component were recruited. Of 82 eligible, 10 agreed to participate. Online interviews were conducted via MS Teams and audio recorded. A photo-production technique (45) was used, asking participants to take photos of places where they felt safe, unsafe, or supported, to anchor discussion. Topics included service experience, perceived mental health impact, relationships, education, and coping (see Table 2). This approach was piloted with PPI group members.

### 2.3 Ethical approval

Ethical approval was secured for the evaluation, reference numbers: ETH2223-0200 and ETH2324-0074. In line with this approval, prior to taking part in any research, all participants were given an information sheet, written in age- appropriate language, and a consent form. The participants were asked to read the information sheet, and if happy to take part in the research, to sign and return the consent form. All participants had the opportunity to ask questions about the research and/or their involvement and were free to withdraw at any point during data collection. For young people that were under 17 years of age, assent to take part in the research was obtained from the young person, in respect of their developing autonomy, and consent was obtained from a parent/guardian. Debrief sheets providing further information about the purpose of the evaluation, contact details for the evaluation lead, and links to further information or support were shared with all participants after interviews were completed.

### 2.4 Analysis

#### 2.4.1 KPI and cost effectiveness analysis

Quantitative KPI data was transferred to the statistical software package (SPSS, v.30), the data were cleaned (removing incomplete data), and within-group repeated measures analyses (primary analysis) was conducted. These included paired sample t-tests, or non-parametric equivalents, and acted as indicators of progress towards outcome delivery on comparison of pre- and post-intervention assessments. A cost analysis was conducted to assess value for money of the intervention, considering the total overall planned budget of £401,961 (according to the commissioning document) broken down to the funding available for the delivery of each intervention across the four intervention components.

Self-reported KPI measures of wellbeing were used as proxies for impact. These included the DIALOG score (22), which, through 11 set questions each on a 7-point scale and across eight life domains, captured young people’s subjective quality of life (with scores possible from 1 to 5 for each domain); and the Wellbeing Star (23) which was used to assess a young person’s perceived ability to ‘live as well as they can’ through a holistic assessment of 8 outcome areas including friends and family, resilience, confidence, and functional wellbeing (with scores possible from 1 to 7 for each outcome area). Cost-effectiveness (value for money) was then estimated by calculating the cost per unit of improvement in each outcome measure. This was done by dividing the total delivery cost of each intervention component by the observed change in DIALOG and Wellbeing Star scores, enabling the calculation of a cost-per-point improvement estimate for each wellbeing domain. For each participant. These scores were they aggregated and averaged for each intervention.

Qualitative KPI and cost effectiveness data from surveys and proformas was analysed to identify themes, using the intervention outcomes as a framework. The qualitative data was then used to cross check and provide depth to the quantitative KPI and cost effectiveness data. The extent to which intervention outcomes were met was assessed through mapping all data across relevant logic model outcomes to facilitate data synthesis amongst data analysts. Audio recordings from interviews were transcribed verbatim and analysed in Delve.io and NVivo using Braun and Clarke’s (24, 25) six-step thematic analysis framework. This involved: (1) familiarisation with the data through repeated reading of transcripts; (2) systematic generation of initial codes; (3) organising codes into potential themes; (4) reviewing themes in relation to coded extracts and the entire dataset; (5) defining and naming themes; and (6) producing the final analytic narrative with supporting quotations. Young people’s interviews were analysed deductively, guided by three pre-specified evaluation questions: experiences of service use, perceptions of ‘active ingredients’ for change, and perceived impacts on wellbeing and relationships. A deductive approach was used here to ensure that the analysis directly addressed the evaluation aims and captured the voices of young people in relation to the intervention’s intended mechanisms of change. The adult interviews and focus groups were analysed using a combined deductive and inductive approach. Deductive coding, again based on evaluation questions, enabled comparison across participant groups, while inductive coding allowed unanticipated issues (such as systemic barriers to implementation and cross-sector collaboration) to emerge from the data. This dual approach was chosen to balance evaluation-driven inquiry with openness to novel insights that could strengthen understanding of the intervention’s context and delivery. Illustrative quotations are presented below to contextualise and substantiate key findings.

## 3 Results

### 3.1 Intervention fidelity, reach and dose

Only seven (54%) of the 13 targeted educational settings engaged with the BR component of the intervention, with varying levels of engagement across those. All seven settings took part in at least two consultancy calls and at least three audits, but only five offered at least one training session or webinar, and four (30%) completed all parts of the intervention. This reduced engagement may have negatively impacted the implementation of learning, and hindered the support available for, and offered to young people. Intervention stream leads suggested that challenges in engagement may have resulted from a disconnect between the aims of the intervention and the systemic and capacity constraints within educational settings to implement such interventions. One participant reflected: “…regardless of what… the evidence base is telling us… being slightly more realistic about the capacity [of schools] and the place that our educational settings are in at the moment.” (Intervention stream lead).

Additional engagement challenges occurred with the CCs, with four hubs planned but only three were formed. However, there did appear to be a wide diversity of young people involved in the three CC that were formed, with 46 ethnically, and geographically diverse young people having engaged directly in the CC, including 12 neurodiverse young people, at the point of evaluation. An additional 250 additional contacts were made with young people across CC outreach activities.

**Figure 2:**
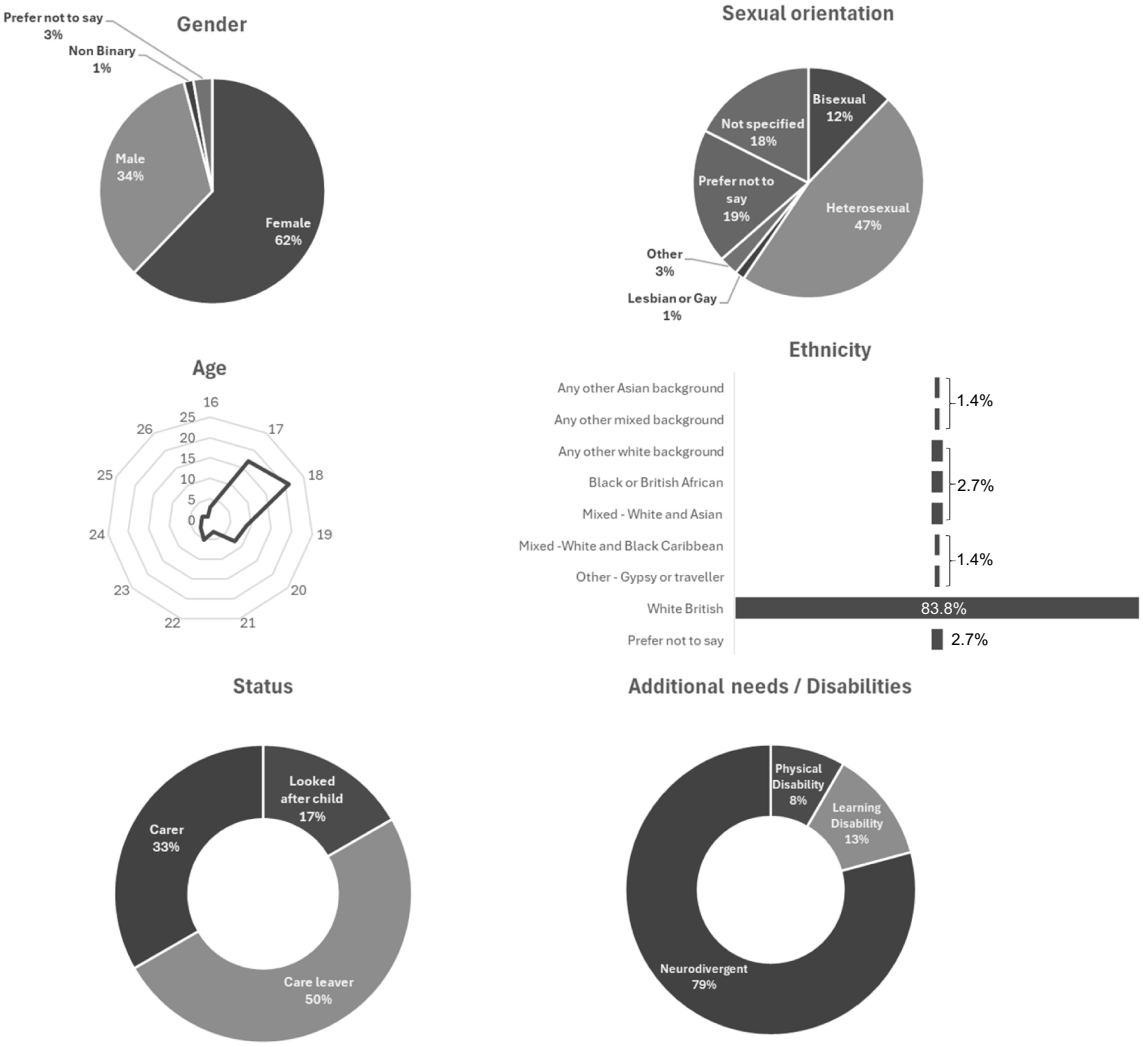
Characteristics of young people engaged in the intervention.

In terms of the WN, many young people that took up the support had legally protected characteristics and lived in areas with multiple deprivation (see Figure 2). However, intervention delivery personnel expressed ongoing concerns about effectively reaching those experiencing the greatest inequalities: “How do we genuinely get to those young people with the most inequalities?” (Intervention stream lead) Another queried: “Where are the young people that really could do with support that we do not know about?” (Intervention stream lead).

The UW component offered professionals (*n* = 707) mental health focused training sessions (*n* = 50). Participants included local government staff (*n* = 248) (including representatives from departments such as adoption, housing, social workers, and the leaving care team), staff from health care (*n* = 43), policing (*n* = 36), voluntary, community and social enterprises (*n* = 80), education (*n* = 94), and community members (*n* = 33).

A lack of coherence of the intervention’s four components may have added to concerns about effectively reaching those experiencing the greatest inequalities, and may have led to inefficiencies and an underutilisation of resources and time: “I did not feel there was a strong tie between any of them … it was by serendipity rather than actually a conscious planned thought-out process that how are all these going to work together?… could have made more efficient and effective use of the funds available if there had have been a bit more of a better thought-out plan.” (Intervention stream lead).

### 3.2 Mechanisms of impact and how change was generated

The WN service was partially effective in improving readiness for life transitions, with statistically and clinically significant improvements in satisfaction with mental health (see Table 3).

**Table 3:** Young people’s Dialog scale scores pre and post intervention.

Measure	Before rating	After rating	Change	Effect size in magnitude of change using Hedges correction
Mental health	2.82	4.88	2.06	1.13
Physical health	4.14	4.58	0.44	1.24
Job/ academic situation	4.73	5.02	1.14	1.65
Living situation	4.76	5.35	0.58	1.43
Satisfaction with leisure activities	4.58	5.32	0.74	1.52
Family satisfaction with relationships	4.73	5.44	0.71	1.37
Satisfaction with friendships	4.76	5.38	0.62	1.53
Satisfaction with personal safety	5.14	6.38	1.24	1.38

All changes were statistically significant at *p* < 0.05.

**Figure 3:**
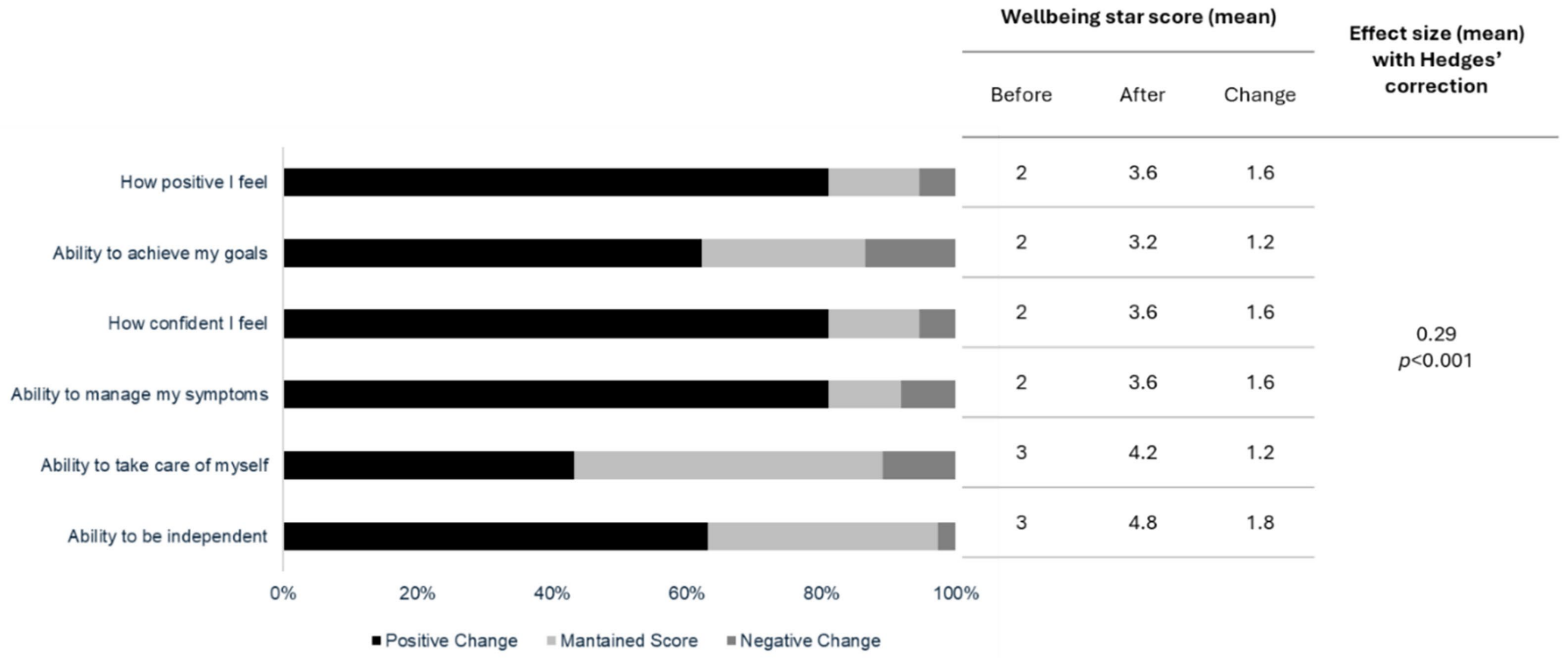
Change in service user Wellbeing Star scores (scale of 1–5) pre/post intervention (*n* = 37).

Most (89%, *n* = 37) young people that attended the WN support reported that the service helped improve their wellbeing, with largest improvements reported in overall mental health, feelings of positivity, confidence, and ability to manage symptoms (see Figure 3).

The focus of the CC and WN intervention components on listening and sharing in a supportive environment often provided relief for young people when individual dialogue was not possible. One young person shared, “…you had this interaction and communication across different people in the group. I utterly realised that, having group conversation becomes very, very helpful…” (Young person 3).

Peer-to-peer support, available through the CC hubs, was also important, supporting healing and building resilience. One young person recalled, “one of the other members also shared her honest feelings… she said it had worked for her so for another person, someone of my age group, to share this information and it worked for her.” (Young person 5).

Over half (60%, *n* = 30) of school staff that took part in the UW component reported that the training could make a difference to their work with young people—boosting staff confidence for effective support delivery, and helping young people in times of change: “The biggest impact the training has had for me is definitely the self-confidence… gaining the additional knowledge to better support the young people that I work with.” (UW participant).

### 3.3 Influence of context on intervention delivery and acceptance

There were good levels of acceptability amongst young people invited to the WN service, with 65.4% (*n* = 102) taking up the service and only 3.8% (*n* = 6) declining the invitation (including those that did not attend). Young people that took up the WN service gave high scores based on enjoyment (9.16/10), their sense of safety (9.54/10), feeling listened to (9.46/10), and how well they felt that the sessions were run (9.37/10).

The opportunity for young people to access support outside of school was highlighted as being important by CC intervention stream leads: ‘it became very apparent that they all just wanted to talk about the pressures of education […] that actually they had a space where they could let that out and then forget about it and be silly and play and remember that they are a child and they are young and it’s okay to play, and all those pressures, they are there, they are not going to go away, but you have to make sure then you have an outlet to exhaust your inner child…’

Creating spaces for storytelling and the sharing of lived experiences was important in fostering engagement and acceptability amongst young people. Sharing personal narratives validated individual experiences and built a collective sense of understanding and community. One staff member reflected, “It’s about making a space where they [young people] can talk about it and those lived experiences are shared and instantly then you feel seen, you feel heard, you feel validated…” (VCFSE representative 1).

The need to recognise challenges faced by specific groups, such as young adult carers, was considered important to make the intervention more accessible and meaningful. One staff member involved in the CC hubs highlighted how proactive communication about caring responsibilities could improve support “…putting out that publicity and that comms around young adult carers… ‘Do you have a caring role?’ […] that has got to have a massive effect on their wellbeing…” (VCFSE representative 2).

To reduce fear and vulnerability and build trust, support should be introduced in a phased or layered manner, particularly for young people hesitant to share their experiences. As one young participant revealed, “…most times that was my first time of sharing my bad experiences… and I was afraid that sharing this information would harm me more.” (Young person 5).

Delivering interventions in settings that resonate with young people’s lives, and differentiation between compulsory (such as schools) and voluntary spaces (a location chosen by the young person), was important to helping those most in need of accessing mental health support. As one staff member described, “your third space is sometimes your most important one because it’s the only one that’s chosen…” (VCFSE representative 1).

### 3.4 Value for money

In the absence of a controlled study, pre- and post- intervention survey data from participants was used as the basis for resource impact analysis. These individuals are described as ‘engaged’, noting this differs from the total number who took part in the intervention (51 young people completed the WN programme, 46 engaged regularly with CC). For the BR component, survey respondents were drawn from across the seven participating schools. Due to low survey response rates, the CC component was excluded from the quantitative cost impact analysis.

The three components delivered low cost per participant improvements, suggesting value for money, affordability and potential for scalability (see Table 4). Cost per engaged participant ranged from £252 to £3,952, with the lowest cost associated with workforce upskilling and the highest cost being associated with the WN workstream.

**Table 4:** Cost per intervention stream, and per engaged participant.

	Budget per intervention stream (£)	Engaged participants (survey respondents) (n)	Cost per engaged participant (£)
Wellbeing Navigators (year 1):	£134,374	34	£3,952
Building Resilience:	£49,500	7 N.B. engaged participants = schools engaged (n)	£7,071 N.B. £ = cost per school engaged
Upskilling the workforce:	£60,000	238	£252
Total budget^*^	£401,961		

* Total budget includes the Community Collaborations intervention stream (£158, 087). However, this intervention has not been included in this cost analysis as there was no survey response data at the point of evaluation.

The cost per unit of improvement in DIALOG scores (i.e., satisfaction with life domains) ranged from £1,919 (for a unit improvement in increased satisfaction with mental health) to £8,928 (for a unit improvement for increased satisfaction in physical health), with the most cost-effective improvements in satisfaction being observed for improvements in cost per unit improvements in relation to increased satisfaction with mental health.

For Wellbeing Star scores, a 10% improvement was estimated to cost between a range of £488 and £919, for the following variables; ‘how positive I feel…,’ ‘ability to achieve my goals’, ‘how confident I feel’, ‘ability to manage my symptoms’, ‘ability to take care of myself’ and ‘ability to be independent’ with the lowest same cost per improvements of £488 for an improvement in positivity, confidence, and symptom management. The highest cost per improvement was associated with the variable of ‘ability to take care of myself’ (£919).

Although the CC component was not included in the quantitative cost analysis, interviews with intervention stream leads highlighted that whilst the number of engaged young people was relatively low, the potential cost benefit of the CC for young people was high: ‘If people look at it just from a perspective of numbers you probably would say no because of the numbers of beneficiaries being engaged with… however I think for the amount of resource, time … to even get to this stage is far and beyond what [they have got] in the grant…’ (Intervention stream lead).

## 4 Discussion

This evaluation offers insights into the delivery of a complex mental health intervention for 16–25-year-olds in the East of England. The intervention sought to offer mental health support to young people, and training to educators, and public health professionals—linking education, health, and the VCFSE sector. The premise of this cross-sector model aligns with evidence that integrated services can improve youth mental health outcomes (26).

Evaluation findings indicate that meeting young people “where they are,” physically and emotionally, is crucial to engagement. The CC hubs “third spaces” helped young people come together, discuss their challenges, and navigate pressures linked to transitions, whilst the WN model, delivered flexibly in community settings, achieved strongest engagement with young people. The relational, low-barrier approach of the WN component enabled service access, and trust building, supporting previous findings on the value of co-located services (27) and relational continuity (28) for young people. For example, Canada’s ‘ACCESS Open Minds’ programme reported the value of flexible, youth-centred hubs that reduce barriers to engagement by embedding services within local communities (29, 30), whilst Australia’s ‘Headspace centres’ found that early intervention in accessible, non-clinical settings, demonstrated improved service uptake and satisfaction among young people navigating education-to-work transitions (31, 32).

The UW component was important to supporting young people going through periods of transition, with training offered to frontline staff across education, health, and community sectors not only expanding the intervention’s reach but also creating opportunities for sustainable impact through capacity building. In contrast, the BR component was constrained by capacity and institutional readiness which reveals how institutional structures may hinder support at transitional stages. Moreover, the pilot of a ‘Whole College approach’ revealed a significant gap in provision for post 16 learners (33, 34), highlighting the need for continued development of mental health support across further education (33). Findings from this evaluation indicate that reaching young people not in education, employment or training (NEET) remains challenging. Structural barriers, such as stigma, low trust, and limited awareness, may have hindered engagement, reflecting previous research assessing the impact of mental health interventions (35). This may have been exacerbated by the lack of integration and cohesion across the interventions four components, which mirrors systemic fragmentation during transitions from child to adult services. It also highlights the importance of tailored, flexible and accessible mental health support for young people. This is particularly important for vulnerable young people such as care leavers (36).

The evaluation findings also revealed that the intervention had a statistically significant positive impact on helping young people going through transition periods to feel more positive and confident and have a better ability to achieve their goals. Such changes are important, considering the role that transitional stresses, such as those associated with becoming self-sufficient and making future-shaping decisions about living situation, education and careers, can have on young people’s mental health and wellbeing (37).

Cost effectiveness is referred to throughout the paper in the broad descriptive sense, referring to whether the multi-component, mental health intervention provided good value for the money spent. In terms of the costs associated with the intervention, the UW component demonstrated good value for money, with cost per engaged participant (£252) comparing favourably to the average cost of a referral to a community young person mental health service which is at least £2,338 (as of 2017) (38), highlighting scalability and potential to generate system-wide benefits. Whilst the WN component was the most expensive aspect of the intervention at £3,952 per engaged participant, it still compares favourably to traditional referrals and has strong engagement, suggesting cost–benefit through earlier intervention and prevention,.

In support, previous economic evidence demonstrates that early intervention and prevention in youth mental health yield significant long-term savings by reducing reliance on specialist services and improving educational and employment outcomes (39, 40).

Other recent studies that have used multi component elements in mental health interventions to support young people have also demonstrated economic benefits.

The HeadStart (Big Lottery Fund, 2016–2023) supported 10–16-year-olds in 6 local authority areas with an area-wide programme that utilised school resilience curricula, digital tools, and targeted one-to-one support, parent/family engagement as compared to the usual local services. This study reported a positive return on investment ranging from £1.80–£2.30 per £1 invested with a reduced demand on CAMHS & school exclusions.

The Children and Young People’s Health Partnership (46) reported on an intervention within integrated primary, community & specialist care settings that included interventions to support mental-health and physical health using digital tools and social prescribing for 0–16 year olds within 6 local authority areas in South London (41). Compared to the usual GP/secondary care pathways, this study demonstrated an incremental cost of £70 per child with improved mental-health scores, with the intervention being considered potentially cost-effective, in the longer term, at a cost per QALY threshold not exceeding £20,000. These studies along with the findings reported in this paper, indicate the potential for multi-component interventions to deliver improvements in health and wellbeing alongside economic benefits.

It is acknowledged that there are some limitations to the approaches we have taken to explore the resource use and economic impact of the intervention alongside the outcome measures. Firstly, we were unfortunately, unable to triangulate any of our economic quantitative findings with those of our qualitative findings to provide any significant ‘qualitative’ evidence or insights into cost effectiveness or value for money. Secondly the cost analysis and value for money approaches taken in this study are distinct to the more formal, quantitative economic evaluation methods used in for example trials, where costs and health outcomes (effects or benefits) of two or more interventions are compared. Costing analyses and value for money assessments are therefore a methodologically weaker form of assessment, though defensible as they offer highly valuable insights into economic impacts for decision makers in the absence of better data from more robust studies (42). As such, the results from the evaluation reinforce calls for policymakers to prioritise community-based and preventative approaches that can reduce fragmentation, improve transitions, and generate both social and economic value, with longitudinal rigorously designed evaluations needed to assess sustained economic impact.

### 4.1 Implications for policy and practice

#### 4.1.1 Addressing gaps in mental health provision at key transition points

Mental health provision must be better aligned with the realities of young people’s lives, particularly at key transition points. Policy frameworks should extend the Whole School Approach beyond schools, embedding mental health promotion and early intervention into colleges (16 + years), universities, apprenticeships, and entry-level workplaces.

#### 4.1.2 Strengthening community-based interventions

Community-based models like WN and CC can provide a safety net for young people who fall between traditional services, including care leavers, LGBTQ+ youth, young carers, and neurodivergent individuals (43). Success lies in relationship building—between staff, peers, and young people—often fostered in non-clinical spaces ‘third spaces’ that support trust and engagement. Creative approaches were especially valuable where communication was limited. While training for teachers, providers and community stakeholders was well received, there were calls for sustained funding to enable ongoing evaluation and adaptation.

### 4.2 Designing future mental health interventions

Future interventions should be co-produced with young people, drawing on lived experience and addressing their broader social and material conditions. Creative, inclusive approaches can reposition young people as storytellers of their own lives, facilitating engagement and ensuring that diverse, marginalized voices shape the design and delivery of services. Using biographically relevant content, tailored to individuals, and groups with diverse experiences and from different backgrounds, rather than clinical diagnoses, may be crucial to better connect with, and support a wider range of young people.

### 4.3 Embedding evaluation and learning

Robust evaluation frameworks are essential not only to assess effectiveness but to understand how, why, and for whom interventions work. This aligns with the MRC guidance on complex interventions. In this evaluation, applying such a framework revealed both the value and limitations of multi-component models, including gaps in integration across strands.

## 5 Conclusion

Place-based interventions, when delivered through cross-sector collaboration and grounded in co-designed, youth-informed practice, have the potential to improve wellbeing and offer good value for money, as seen through the promising outcomes of the WN and UW strands. These components also showed the importance of trust, flexibility, and embedding services in spaces that young people choose to access.

However, the intervention also revealed ongoing challenges in engaging some groups of young people, underscoring the importance of tailoring support and communication using biographically relevant and culturally responsive narratives. Crucially, young people must be meaningfully involved in the design and delivery of services. However, building these models requires trust with communities, time, and sustainable investment to ensure they are responsive, inclusive and impactful.

## Data Availability

The data that support the findings of this study are available on request from the corresponding author. The data are not publicly available due to privacy or ethical restrictions.

## Author disclaimer

The views expressed are those of the author(s) and not necessarily those of the NIHR or the Department of Health and Social Care. For open access, the authors have applied a Creative Commons Attribution (CC BY) licence to any Author Accepted Manuscript version arising.
