# Supplementary material for: Gaps and opportunities in mental health support for young people: a process evaluation of a multi component intervention
Source: Front Public Health. 2025 Nov 5;13:1684562. doi: 10.3389/fpubh.2025.1684562 (PMC12626782; doi:10.3389/fpubh.2025.1684562)
Supplement: Supplementary file 1 [file supplementary_material.DOCX]

# **Appendix A: KPI data collected for quantitative analysis**

Wellbeing Navigators (MIND)

- Number of young people referred to Wellbeing Navigator service
- Outcome of referrals
- Number of young people (YP) supported by wellbeing navigators
- Demographics of YP supported by wellbeing navigators
- Service user feedback
- Has the wellbeing navigator service increased the ability to be independent for service users? (Resilience proxy?)

Building resilience (Young Minds)

- School college audit results: survey for adults; half day insights for YP; trauma-informed principles
- Training & Services measures: confidence & practical next steps

Community Collaborations

- Number of YP accessing the service / collaborations
- Duration of engagement
- Activities / collaborations engaged in
- Demographics of YP supported by the collaborations
- Has accessing support from organizations within the collaborations increased the wellbeing of the service user?
- What impact has the service / project had on the service user?

Upskilling the workforce

- Feedback from participants on course:
- Please rate your level of knowledge prior to this course (1 being low and 5 being high)
- Please rate your level of knowledge at the end of this course (1 being low and 5 being high)
- Pease tell us about the most/least useful aspects of the training – and how you will use what you have learnt in practice.
- Have you identified any further learning and development needs?
